# Job Dissatisfaction Among Elderly Care Nurses Working in Hospital Inpatient Settings: A Qualitative Study

**DOI:** 10.1177/23779608261478192

**Published:** 2026-08-24

**Authors:** Vilma Žydžiūnaitė, Marija Truš, Indrė Brasaitė-Abromė

**Affiliations:** 1 Nursing Department, Faculty of Health Sciences, 87241 Klaipėdos Universitetas/Klaipėda University, Klaipėda, Lithuania

**Keywords:** geriatric nursing, elderly care, inpatient care, nursing workforce, job dissatisfaction, role overload, latent qualitative content analysis

## Abstract

**Introduction:**

A knowledge gap exists regarding how physical, psychosocial, and organizational stressors interact dynamically over time to drive nurse dissatisfaction. This qualitative study explores the lived experiences of hospital inpatient elderly care nurses navigating their institutional environment. The aim was to uncover the specific operational dynamics of job dissatisfaction and provide an empirical model of these cyclic, self-reinforcing patterns.

**Methods:**

A qualitative research design was applied for the study. The purposive sample consisted of 21 nurses; the sample size was determined by data saturation. Data were collected via semi-structured interviews and analyzed using latent qualitative content analysis.

**Results:**

A cyclic model was developed to delineate four components of nurse dissatisfaction in hospital inpatient elderly care, where an unfavorable practice environment shapes work and role overload, which subsequently influences physical and psychosocial exhaustion. Such exhaustion leads to professional distress when clinical realities conflict with professional expectations, ultimately looping back into the system to compound the operational cycle.

**Conclusion:**

The developed interpretive model provides experience-based insights for nursing leadership to target the cyclic feedback mechanism through supportive structures and active modifications to the daily elderly care nursing environment. These qualitative findings show that the retention of elderly care nurses involves comprehensive rather than isolated interventions.

## Introduction

The global healthcare landscape faces challenges in nursing workforce sustainability, with hospital inpatient settings exhibiting elevated rates of occupational distress and turnover (Hayward et al., 2016; WHO, 2020). Job dissatisfaction among nurses in hospital inpatient elderly care is a multifaceted systemic issue characterized by disengagement and emotional exhaustion driven by high work demands, inadequate staffing, and a lack of organizational support that compromises the delivery of consistent, patient-centered care (Abdi et al., 2019; Kang & Bang, 2024; Marinho et al., 2022; Senek et al., 2020).

## Review of Literature

Intensive workloads force nurses to prioritize documentation over direct patient interaction, creating a sense of professional and ethical inadequacy (Dasgupta, 2013; Foong et al., 2021; Kang & Hur, 2021; Kumlin et al., 2020; Tuckett et al., 2014). These operational imbalances cause higher rates of dissatisfaction than alternative clinical environments (McHugh et al., 2011; Zhang et al., 2022). Physically, long shifts, night work, and manual patient lifting cause chronic musculoskeletal issues that diminish long-term work sustainability and health (Ge et al., 2021; Harrad & Sulla, 2018; Jakobsson Larsson et al., 2024; Li & Chen, 2023; Li et al., 2024; White et al., 2019). Psychosocially, the workplace is constrained by high patient-to-nurse ratios, mortality exposure, poor teamwork, limited managerial support, and workplace violence (Galanis et al., 2023; Harrad & Sulla, 2018; Wudarczyk et al., 2025). These difficulties are exacerbated by the emotional labor of managing advanced cognitive decline, family expectations, and professional composure under pressure (Dasgupta, 2013; Kim & Lee, 2020; Senek et al., 2020; Wang & Nguyen, 2023; Wudarczyk et al., 2025).

The qualitative approach allowed nurses to express the emotional burden of elderly care and highlighted region-specific factors. The eligibility criteria established a homogenous, specialized cohort for deep insights into job dissatisfaction dynamics (Elo et al., 2014). Selecting university-educated nurses with over a decade of specialized experience isolated systemic and operational drivers, minimizing confounding variables like entry-level transition shock or differing educational tracks. This focus supports transferability by offering a context-specific model for comparable healthcare settings.

Several limitations must be noted. Excluding nurses with diplomas from non-university higher education institutions (kolegijos) leaves the experiences of those professionals unexamined. Similarly, requiring a minimum of 10 years of clinical experience excluded early-career nurses, leaving entry-level attrition and transition shock unexamined. Socioculturally, the historical undervaluing of the nursing role as subordinate to medicine, coupled with a weak research culture and low prestige, can hinder open discussion. Finally, the exclusively female composition presents limitations regarding gender bias and transferability. Nursing in Lithuania remains female-dominated, with men constituting less than 1% of the workforce, intertwining the discipline with traditional societal expectations of caregiving and historical post-Soviet occupational structures (Gribačiauskaitė & Žilinskienė, 2024; Karosas & Riklikiene, 2008). This sample reflects national demographic realities rather than restrictive study criteria, meaning the findings may not represent the experiences of male nurses or be broadly transferable to gender-diverse healthcare systems (Gribačiauskaitė & Žilinskienė, 2024).

The research aim was to uncover the physical, operational, and psychosocial dynamics driving job dissatisfaction, providing an empirical model of these cyclic, self-reinforcing patterns within hospital inpatient elderly care. To achieve this aim, the following research questions were addressed: “What specific, daily tasks do elderly care nurses perceive as related to job dissatisfaction? How do elderly care nurses in hospital inpatient settings describe their lived experiences of job dissatisfaction?”

## Methods

### Research Design

This study used semi-structured interviews and latent content analysis to explore the underlying causes of job dissatisfaction among elderly care nurses (Choi et al., 2025). A qualitative approach is ideal for examining the complex, personal dimensions of nursing experiences within hospital inpatient settings (Gao et al., 2025; Renjith et al., 2021).

### Participants and Settings

The study employed purposive sampling to recruit twenty-one nurses working in inpatient elderly care across Lithuania’s five largest cities: Kaunas (n=4), Klaipėda (n=5), Panevėžys (n=4), Šiauliai (n=4), and Vilnius (n=4). The sample consisted of female nurses, reflecting broader national demographics where women comprise over 95% of all nursing professionals; male nurses in elderly care are not explicitly identified in official statistics (Institute of Hygiene, 2025). Therefore, gender was not included in the sample selection criteria. To ensure an experienced and structurally homogenous sample, participants were selected based on the following eligibility requirements:

• Inclusion criteria: (i) professional role: licensed nurses currently working in hospital inpatient elderly care settings; (ii) geographical distribution: employment within an inpatient facility located in one of Lithuania’s five largest cities; (iii) clinical experience: a minimum of 10 years of total hospital inpatient experience; (iv) specialized experience: a minimum of 10 years of specific experience dedicated to elderly care settings; and (v) educational attainment: possession of a university-level Bachelor’s degree in nursing or higher.
• Exclusion criteria: (i) primary practice setting: nurses whose clinical experience was primarily rooted in primary healthcare settings rather than inpatient hospital wards; and (ii) educational background: nurses who completed their education at a college (a non-university higher education institution in Lithuania) and held a professional bachelor’s degree instead of a university degree.

The sample consisted of 21 participants, determined by inductive category saturation (Saunders et al., 2018). Participants had a mean age of 45.52 years and a mean total hospital experience of 20.43 years, with 17.39 years specifically in elderly care. All participants held a bachelor’s degree in nursing, and nine also held a master’s degree.

To ensure rigor, sampling continued until thematic saturation was reached, defined as the point where successive interviews ceased to yield novel codes or themes (Rahimi & Khatooni, 2024; Sirvan, 2025). Preliminary saturation was achieved by the 17th interview. Four additional validation interviews (18–21) produced no new insights, confirming that further recruitment was redundant and concluding the sample at 21 participants (Laari, 2025).

### Data Collection

Data were collected between December 2024 and May 2025 through 21 semi-structured interviews. All sessions were conducted in Lithuanian and held in participant-selected settings to foster rapport and encourage open disclosure (Sirvan, 2025). Prior to each interview, researchers explained the study aim, questions, and practice value. Interviews were audio-recorded, transcribed, and analyzed sequentially. Seventeen interviews (RN3–13, RN15–17, RN19) were conducted online, and four (RN1–2, RN14, RN18, RN21) were face-to-face. Interview durations ranged from 56 to 194 minutes.

Data collection was collaboratively executed by all three members of the research team, who are independent nursing scholars trained in qualitative methodology. Distributing the interviews across the full team minimized individual interviewer bias and enhanced reflexive interpretation. To maintain methodological consistency, researchers compiled field notes immediately after each session. The team also held regular debriefing sessions after every 3 to 4 interviews to co-examine new transcripts, compare emerging narrative patterns against the established framework, and ensure consistent deployment of follow-up and probing questions.

### Tool

The interview utilized a framework consisting of introductory, probing, and follow-up questions to trace temporal processes (see Table 1). To maintain consistency while allowing for exploration, participants shared specific clinical experiences, discussed patient or family cases, explained professional decisions, and reflected on the meanings of those experiences.

**Table 1.** Semi-structured interview tool questions

Introductory questions	Follow-up questions
1. Can you describe a recent situation at work that made you feel frustrated, tense or unsupported?	What was going through your mind at that moment?
2. When you think about your daily work, what aspects of your role cause you the stress?	What did you do in response to that feeling?
3. Describe a time when you felt you couldn’t provide the quality of care you wanted to. What was happening?	Why do you think that situation made you feel that way?
4. What makes you think about difficulties in your current position in elderly care while working in inpatient unit at hospital?	How did that experience make you feel about your role as a nurse?
5. How does the work culture and management on your unit influence your day-to-day satisfaction?	Can you help me understand that a little better?

*Note*. Developed and compiled entirely by the authors.

As illustrated in Table 1, the interview protocol used a two-stage framework:

• Introductory questions (Socio-operational reality): Questions 1 through 5 elicited narrative descriptions of daily practice. They progressed from specific incidents, such as unsupported situations (Question 1) and compromised care quality (Question 3), to broader structural reflections on systemic stressors, organizational positioning, and unit management culture (Questions 2, 4, and 5) to ground the conversation in everyday routines.
• Follow-up and probing mechanisms (Latent meaning extraction): Probes were deployed dynamically rather than rigidly to systematically unpack four levels of latent processing:
• Cognitive processing: “What was going through your mind at that moment?”
• Behavioral coping adjustments: “What did you do in response to that feeling?”
• Attributional and root-cause reflection: “Why do you think that situation made you feel that way?”
• Professional identity and role erosion: “How did that experience make you feel about your role as a nurse?”

The tool was designed based on the qualitative interviewing frameworks of Brinkmann and Following Kvale (2015), the interview protocol utilized a two-tiered questioning strategy to generate descriptive and reflective depth (Table 1):

• Constructing the manifest anchor: Introductory questions prompted participants to reconstruct concrete events, such as missed care or structural difficulties, providing the descriptive foundation for the interview.
• Latent extraction: Follow-up questions served as the primary analytical drivers to explore meanings. Probes regarding the nurse’s mindset, coping mechanisms, and professional self-image transitioned the text from a timeline of daily tasks into a reflective narrative.

The semi-structured interview guide was developed and validated through a multi-stage framework to minimize instrument bias and ensure sufficient descriptive detail for conceptual model generation.

Content validation was completed via external expert review by 3 professors specializing in geriatric nursing and qualitative methodologies (Polit & Yang, 2015). Following qualitative tool guidelines (Kallio et al., 2016), the panel independently evaluated the introductory and follow-up questions for:

• Exploratory scope: confirming questions were broad enough to capture the physical, operational, and psychosocial dynamics of job dissatisfaction without introducing preconceived frameworks.
• Contextual appropriateness: confirming terminology accurately reflected the daily clinical conditions of hospital inpatient elderly care.

Based on the review, the panel’s consensus feedback was used to refine 2 introductory questions, eliminating leading phrasing and optimizing their open-ended structure to maximize spontaneous, reflective data.

Face validation and pilot testing: to establish face validity, a pilot test was conducted via cognitive debriefing interviews (Bengtsson, 2016) with 2 eligible practicing hospital inpatient nurses. These participants were excluded from the final study sample (N=21). The pilot sessions evaluated comprehensibility, ensuring the distinction between operational and reflective prompts was clear, and linguistic accessibility, confirming nurses could articulate exhaustion without feeling judged. Feedback demonstrated that the guide flowed logically from everyday tasks to reflective inquiries. Because the questions elicited the necessary descriptive data for inductive analysis, no structural or wording modifications were required before data collection commenced.

### Data Analysis

Latent qualitative content analysis was used to interpret the implicit meanings within the textual data, serving as an interpretive process to develop subcategories and categories (Kleinheksel et al., 2020). Following Graneheim and Lundman (2004), analysis involved six steps: data preparation, immersive familiarization, identifying meaning units, developing latent codes, grouping codes into subcategories and categories, and interpreting underlying patterns.

To preserve original meanings, cultural nuances, and idiomatic expressions, qualitative analysis was conducted entirely in Lithuanian, with English translation occurring only for final representative quotations during manuscript drafting (Van Nes et al., 2010). Subcategories and categories were developed directly from the native text. To ensure linguistic accuracy, semantic equivalence, and academic neutrality, the translation process followed a rigorous protocol:

• Initial translation: selected Lithuanian quotations were translated into English by the primary research team.
• Independent review: an independent bilingual linguist checked the English segments against original transcripts to verify conceptual equivalence.
• Discrepancy resolution: phrasing differences were resolved collaboratively by the team to maintain the participants’ authentic voices and ensure grammatical accuracy.

Data tracking, coding, and category reduction were performed manually. While computer-assisted qualitative data analysis software serves as an established tool for organizing datasets, the research team printed the physical transcripts to facilitate a collaborative, line-by-line reading process. Individual codes and meaning units were highlighted, extracted, and organized across physical matrix grids (Maher et al., 2018). This manual approach supported iterative cross-examination by the three scholars, allowing the final subcategories and categories to be developed directly from the experiences described by the inpatient elderly care nurses.

The structural architecture of the model was developed using Graneheim and Lundman’s (2004) content analysis framework. First, transcripts were divided into meaning units, condensed, coded, and sorted into manifest subcategories. These subcategories were aggregated into five distinct categories: Navigating work overload, Confronting role overload, Enduring an unfavorable practice environment, Surviving physical exhaustion, and Bearing psychosocial exhaustion. Second, axial sorting was applied to trace the intersections among these categories, showing that quantitative task volumes and qualitative boundary tensions occur concurrently during a shift and combining them into a single component (Stage II: The Drivers). Finally, the operational pathways were linked sequentially to map how job dissatisfaction connects back to the baseline practice environment, reducing the five original categories into a four-stage cyclic structure (Table 2).

**Table 2.** Operational phases of the inductive data processing and model construction

Operational phase	Analytical step	Physical action applied to empirical data
**Phase 1: Manifest Coding and Categorization**	*1. Extraction*	Isolated textual segments containing explicit statements regarding workplace stressors or clinical strains from the raw interview files.
	*2. Condensation*	Shortened the extracted statements to reduce overall word volume while retaining the literal descriptive terms used by participants.
	*3. Coding*	Labeled the condensed statements with micro-level conceptual tags to catalog individual operational and environmental pressures.
	*4. Subcategory sorting*	Grouped codes sharing identical descriptive features into manifest subcategories.
	*5. Category aggregation*	Combined the subcategories into a flat taxonomy of five separate categories representing distinct areas of nurse difficulty.
**Phase 2: Sequential and Structural Mapping**	*6. Operational intersection*	Traced the temporal alignment of the categories during a standard shift, documenting that work overload and role overload occur simultaneously.
	*7. Category reduction*	Merged the two simultaneous workload categories into one operational component labeled as Stage II: The Drivers.
	*8. Loop formatting*	Connected the operational components sequentially based on participant timelines, linking the final category back to the initial stage.

*Note*. Developed and compiled entirely by the authors.

### Rigor

To ensure trustworthiness, the research team used an audit trail, systematic analysis, investigator triangulation, reflexivity, member checking, thick description, and analytical memoing (Lyhne et al., 2025). To minimize participant burden, full raw transcripts were not returned; instead, member checking was performed by sending the formulated subcategories, categories, and selected Lithuanian quotations back to participants (Birt et al., 2016; Elo et al., 2014). Participants confirmed that these findings accurately represented their lived experiences before final English translations were executed. Participants did not review the final abstract conceptual model, which was synthesized by the research team to minimize burden while keeping the framework grounded in the collective data.

Data collection and analysis occurred concurrently to monitor saturation. Inductive category saturation was identified by the 17th interview, as the coding matrix stabilized and no new subcategories emerged. To validate this stability, four additional interviews (18–21) were conducted. Because these final four transcripts yielded no new analytical insights, recruitment concluded at 21 participants (Ahmed, 2024; Rahimi & Khatooni, 2024). Regular team discussions and consensus established a clear audit trail, ensuring the final model reflects the collective data rather than individual researcher bias, thereby enhancing dependability and credibility (Graneheim & Lundman, 2004). Negative case analysis was also incorporated by actively searching for data segments or perspectives that deviated from emerging patterns, refining the cyclic loop model to capture the data’s complexities (Patton, 2015). Finally, to ensure reporting transparency, the study adhered to the Consolidated Criteria for Reporting Qualitative Research (COREQ) checklist.

### Reflexivity Statement

In alignment with latent qualitative content analysis, the research team maintained critical reflexivity to mitigate bias (Graneheim & Lundman, 2004). The team comprised three female academic nursing scholars with doctoral degrees whose activities were confined to higher education and research. None held clinical or managerial roles within the participants’ facilities, which minimized power imbalances and eliminated institutional coercion.

To prevent the researchers’ backgrounds as former clinical nurses and current educators from biasing the data with preconceived frameworks, the following reflexive strategies were implemented:

• Independent coding: initial open codes were created independently by each researcher for the first five transcripts.
• Peer debriefing: structured sessions were held after every three to four interviews to challenge and re-evaluate interpretations.
• Bracketing: post-interview field notes were compiled to log emotional reactions and consciously bracket personal feelings from analysis.
• Audit trail preservation: step-by-step documentation mapping raw meaning units to final categories was preserved for team validation.

### Research Ethics

The study was approved by the Research Ethics Committee of Klaipėda University (Protocol No. 9, 2024-09-18, approval No. 2024-09-107) and conducted in accordance with the Declaration of Helsinki (2024). All participants provided written informed consent prior to data collection. Participation was completely voluntary and independent of employing institutions, with freedom to withdraw at any point without penalty. To ensure confidentiality and anonymity, all identifying information—including individual, facility, and location names—was replaced with numerical codes or pseudonyms immediately after transcription (Saunders et al., 2015; Tsai et al., 2016). Transcripts were stored on secure, password-protected servers accessible only to the research team.

Because the researchers held no clinical or managerial roles within the participating facilities, there were no institutional hierarchies or power imbalances, enabling participants to speak openly without fear of professional consequences. Analytical disagreements regarding code assignments or category boundaries were resolved through consensus during peer-debriefing sessions. The team collectively reviewed disputed transcript segments against raw meaning units until a mutual agreement was reached on refining, merging, or renaming codes. This collaborative process minimized individual bias, preserved an audit trail of interpretive shifts, and supported the overall dependability of the findings.

## Results

Following the qualitative content analysis, data regarding nurses’ job dissatisfaction in elderly care were categorized into five dimensions: work overload capturing quantitative temporal constraints, role overload targeting qualitative functional boundary shifts, practice environment reflecting systemic organizational deficits, physical exhaustion isolating somatic bodily strain, and psychosocial exhaustion addressing affective relational depletion (see Table 3).

**Table 3.** (Sub)categories indicating nurses’ job dissatisfaction in elderly care

Category	Subcategory	Category	Subcategory
**Navigating work overload**	Volumetric patient unpredictability	**Surviving physical exhaustion**	Continuous high-intensity physical exertion
	Institutional personnel deficits		
	Compounded administrative documentation volume		Hazardous manual patient handling
	Shift schedule instability		Prolonged adverse static postures
	Rest period insufficiency		
	Ambiguous operational task protocols		Chronic cumulative musculoskeletal strain
	Fragmented team task coordination		
**Confronting role overload**	Non-reciprocal institutional accountability	**Bearing psychosocial exhaustion**	Sustained hyper-vigilance
	Cognitive compression under multi-task demands		Compassion fatigue from exposure to suffering
	Compulsory absorption of auxiliary and non-nursing tasks		Vulnerability to uncontrolled patient outbursts
	Compensatory performance mon storing of unready peers		Cumulative grief from resident mortality
**Enduring an unfavorable practice environment**	Structural managerial alienation		
	Effort–reward asymmetric discrepancy		Moral distress from fragmented patient interactions
	Workplace ergonomic deprivation		

*Note*. Developed and compiled entirely by the authors based on empirical study findings.

### Navigating Work Overload

Elderly care nurses perceive work overload as a subjective strain shaped by high task volumes, time poverty, staffing constraints, and organizational friction. Job dissatisfaction arises from these temporal limitations and structural work-rest imbalances. Nurses characterized this quantitative workload as unpredictable (RN1-2, RN14), unbearable (RN3, RN7-8, RN9), and exceptionally high (RN4, RN6, RN9, RN15, RN19), particularly during night shifts when patient-to-nurse ratios peak. Continuous working hours without task variance create an unmanageable burden of care:

There is a high workload for nurses, especially at night, with one nurse caring for 30 elderly patients, sometimes less, sometimes more. (RN1)

It's a really heavy workload, because we usually work around the clock, long hours, and you never know in which shift you will have more work to do. I don't even think about the fact that there will be less work. (RN6)

This volume-based stress is exacerbated by a shortage of both nurses (RN3, RN9, RN10, RN16, RN21) and nursing assistants (RN1, RN5, RN8, RN14, RN20). Participants perceived that this deficit reduces the time available per patient (RN3, RN10-19), forcing them to complete daily professional tasks in a constant rush that increases the risk of clinical errors:

There is not enough time to complete professional tasks. Sometimes when there are fewer staff on a certain day, it immediately becomes more of a burden. (RN5)

There are stressful situations when you don t have enough time to complete tasks, so you do everything in a hurry and of course, then mistakes are inevitable. (RN19)

The workload is extended by repetitive documentation requirements that consume shift hours and reduce time for patient care (RN2-3, RN13-21). Participants noted that transferring identical clinical information across record-keeping systems diverts their presence from the bedside (RN4, RN12):

To do everything neatly, correctly and thoroughly, it takes a lot of time for detailed descriptions, and transferring all information into documentation requires a lot of additional time. (RN12)

The volume of writing is immense; we waste precious hours detailing repetitive descriptions on charts when our physical presence is urgently needed by the bedside. (RN4)

Work overload extends beyond single shifts, with participants describing schedule instability, adjustments (RN4, RN6, RN19), and short-notice call-ins (RN10-11, RN13-17) as factors that interrupt personal rest (RN3-11). Nurses reported that these structural scheduling patterns tie into a cumulative sense of exhaustion, which complicates recovery before subsequent shifts begin (RN2, RN12):

Instability of work schedules, frequent adjustments, and being called to work outside of the schedule mean rest, personal leisure, and planned activities suffer. This becomes a constant companion of dissatisfaction because you never know what will happen. (RN2)

When they constantly call you in on your day off to cover a vacancy, the shift work disorder sets in, and you can never fully recover before the next heavy shift begins. (RN12)

Research participants expressed opinions regarding how the continuous, unstructured nature of twenty-four-hour rotations exacerbates physical depletion when nurses are denied recovery periods due to unrelenting task loads and poorly spaced rest windows (RN 1-14):

The work is most disorganized when I have to work around the clock, then I have few days off, and then I can’t rest and come to work tired, which is related to my dissatisfaction with my job. (RN3)

We are on our feet for twelve or twenty-four hours straight with no real breaks, and then we have to return to the floor just as exhausted, which completely drains our physical stamina. (RN7)

This temporal strain is intensified by task ambiguity and fragmented organizational processes. Participants perceived that poorly defined clinical procedures create operational inefficiencies, transforming routine procedures into prolonged, disorganized tasks that drain limited clinical time (RN11-16, RN17-19):

Sometimes there are ambiguities regarding tasks, which then lengthens the time required to complete them, which is already sorely lacking. (RN11)

When it is unclear who handles a specific care process, everything stalls, and we end up losing hours repeating steps or waiting for clarifications on basic tasks. (RN16)

Participants shared how diminished teamwork increases physical and temporal demands. Limited collaborative assistance requires nurses to expend additional time and physical effort, compounding the workload. This challenge arises when rigid task boundaries prevent colleagues from helping with patient transfers (RN1-7, RN15-18):

“Lack of teamwork in turning and lifting patients. There is a shortage of nursing assistants, so colleague nurses refuse to help each other in a patient move because they believe it is only the nursing assistants’ responsibility. (RN4)

Information is contained within a specific profession—nurse-to-nurse only, rather than shared with physicians, therapists, and social workers, resulting in incomplete care for complex geriatric needs of elderly patients. (RN7)

### Confronting Role Overload

Elderly care nurses experience role overload during understaffed afternoon and night shifts, expanding professional boundaries and shifting their experience from collaborative care to isolated decision-making (RN1-4, RN12-16, RN18-21, RN2-17). This isolation contributes to stress as nurses assume ethical and legal accountability without immediate institutional backup, making logistical challenges secondary to the perception of sole responsibility (RN9-17, RN2, RN5-12, RN2-16):

This is especially dominant at night, when you remain solely responsible for all problems, decisions, and actions taken (RN6). When a critical situation happens on a night shift, there is no supervisor or medical team upstairs to ask; you are completely on your own with a human life in your hands. (RN16)

Time constraints fragment the nursing role, blocking practice according to professional standards. Nurses manage competing, simultaneous patient needs, limiting their clinical focus (RN12-19). Participants described anxiety induced by this time poverty, noting a conflict between professional standards and an operational reality that compromises patient safety. Adequate time is unavailable to complete tasks during day and night shifts, particularly during low staffing that alters the scope of practice (RN3, RN5, RN10-19):

You are constantly rushing between five urgent clinical tasks at once, knowing that if your focus slips for even one second while preparing medication in a hurry, the patient pays the price. (RN11)

Participants noted that professional boundaries blur when elderly care nurses absorb low-skill auxiliary and administrative tasks due to a lack of support staff (RN5-8, RN2-17). This role dilution expands the nursing scope into unrelated disciplines, distancing nurses from patient care. Consequently, non-nursing responsibilities, including cleaning patient areas, managing food trays, and transport duties, intersect with their experiences of job dissatisfaction (RN4-7, RN14-20):

Performing tasks typically done by other professionals, such as providing dietary advice (dietician), specialized physiotherapy. (RN14)

Replenishing charts, answering non-clinical phone calls, scheduling appointments not related to patient care, and filing records. (RN20)

Participants noted that lateral role strain occurs when elderly care nurses assume an informal supervisory role to back up colleagues perceived as underqualified (RN1-21). This operational burden requires nurses to monitor, correct, and absorb the uncompleted duties of others to maintain safety. Nurses describe difficulties carrying out professional tasks as they step outside their assigned roles to manage these external performance gaps (RN2-4, RN7-11, RN15-17, RN2-19):

Due to the incompetence of my colleagues, I need to cover their duties by taking over tasks the incompetent nurse cannot perform, leading to excessive workload. (RN7)

### Enduring Unfavorable Practice Environment

Elderly care nurses describe facing an unfavorable practice environment characterized by organizational dysfunction, a lack of institutional support, and structural deficiencies. Participants perceive that job dissatisfaction extends beyond physical and temporal demands, embedding itself in the psychological and material reality of the workplace (RN3-16).

Regarding managerial alienation, participants noted experiencing operational isolation due to an unsupportive management structure. Nurses described a workplace culture where department heads are perceived as operationally removed from frontline care, leaving staff to navigate clinical dilemmas independently (RN8-21). This disconnection is paired with an authoritarian supervisory approach, where management fails to validate quality care while labeling reported operational hurdles as minor (RN3-9, RN11-14). Nurses expressed feeling disrespected and unsupported, noting that managers appear to lack awareness of the resources required to sustain care (RN2, RN4-6, RN9, RN12, RN15-21):

The management presence is missed. When expressing problems, the managers' perception of them is not always adequate; to them, they seem insignificant. But for employees who face those 'small' problems every day and every week, they grow into big problems. Nothing changes. This causes a lot of stress and dissatisfaction with the practical environment. (RN2)

They never asked if the shift was ‘light’ or ‘tough,’ what we need to feel better and provide better care, or what they could do to support us. It is a bossy, authoritarian attitude. There is no empathetic or sensitive work culture. (RN17)

Participants described an effort–reward discrepancy, noting that their heavy operational output and expanding clinical competencies are met with low salaries that fail to reflect the difficulty of their positions (RN1-11). This disparity leaves staff feeling undervalued by the institution, as their contributions do not translate into basic financial security, directly driving job dissatisfaction (RN4-21):

The salary does not match my workload, effort, experience and competence. (RN4)

The salary for the job is not such that we can live with dignity. (RN9)

Participants described a lack of physical tools and material deficits as evidence of ergonomic deprivation within the practice environment. Nurses perceived that management does not secure essential equipment needed to move, turn, or assist immobile elderly patients (RN15-18). This lack of supportive mechanical devices requires nurses to exert their own bodies to meet care expectations, resulting in physical strain (RN 3-7):

There is a lack of tools and equipment for working with immobile and non-walking patients. We are physically exhausted, and department heads don't see the need to take care of the essential tools that will make us healthier and patients happier. (RN3)

Lifting and transferring heavy patients manually every single day without lifters leaves us with chronic back pain. Leadership completely disregards our physical health by ignoring our repeated requests for essential ergonomic tools. (RN5)

### Surviving Physical Exhaustion

Nurses described physical exhaustion as a weariness and bodily depletion tied to high manual labor, continuous musculoskeletal strain, and prolonged static standing (RN1-21). This bodily toll shapes daily realities (RN2-4), involving a heavy volume of hands-on physical interventions required to manage high-dependency, co-morbid patients. Daily routines are dominated by strenuous, repetitive tasks performed consecutively, including managing severe incontinence, executing complex wound dressings, and performing complete bed-baths for immobile individuals (RN2-7, RN15-21). The accumulation of these interventions strains baseline physical capacities and erodes bodily stamina (RN11-20):

Managing complex, co-morbid patients requires frequent, physically demanding interventions, such as complex wound care, bathing, and managing incontinence all contributing to the physical exhaustion of nurses in the daily care of elderly patients. (RN18)

Caring for elderly patients with multiple illnesses, as it requires a lot of physical effort and often leads to fatigue. (RN20)

Accumulating care responsibilities forces nurses into a state of persistent physical exertion without opportunities for recovery, depleting stamina and causing severe exhaustion due to complex patient pathologies (RN1-4, RN8-10, RN14-19, RN19-21). Routine tasks like moving heavy equipment, transporting non-ambulatory individuals, and managing patient care require continuous muscular force that strains baseline physical capacities (RN2-4, RN11-18, RN13). Furthermore, maintaining awkward, fixed postures during extended bathing, transfer, and grooming sequences leads to static strain, muscle fatigue, joint stiffness, and chronic localized pain (RN1-13, RN8-10, RN14-19):

Assisting patients with bathing, where nurses have to stand in one spot for long periods, often in a warm, humid environment, causing significant pressure on the lower back, hips, and knees. (RN1)

Helping patients dressing or assisting with grooming often involves standing in a slightly bent, awkward position. (RN14)

Static postural loading restricts blood flow, accelerating tissue fatigue, while standing immobile in humid care spaces increases thermal load and joint pressure (RN2-18). Maintaining fixed angles causes structural skeletal compression as demanding as active lifting (RN3-9, RN14-21). Consequently, the cumulative impact of manual handling and deep bending leads to long-term musculoskeletal discomfort, persistent back pain, and systemic physical depletion (RN1-5, RN2-7, RN11-21, RN19):

Musculoskeletal strain arises when patients are inactive. Constant and never-ending manual handling related safely lifting, pushing, pulling, and supporting patients to assist with mobility. (RN19)

Nurses lift, reposition, and transfer bedbound or mobility-impaired patients on their own, leading to chronic back pain. (RN3)

### Bearing Psychosocial Exhaustion

Nurses described psychosocial exhaustion as emotional depletion shaped by continuous emotional labor, patient distress, and challenging interpersonal dynamics (RN1-21, RN2-4). Maintaining constant mental alertness to monitor a fragile geriatric population and anticipate unpredictable clinical shifts introduces persistent psychological pressure and exhaustion (RN2-5, RN7-9, RN18-21, RN2-19):

The fear that a patient will fall on my shift is a constant shadow; you can never completely relax your mind because you are checking on them continuously. (RN2)

Patient safety is stressful because we are responsible for ensuring that nothing happens to them, but everything happens, including falls and slips. (RN19)

Daily engagement with degenerative geriatric decline causes emotional fatigue and helplessness when standard protocols cannot alter the clinical course (RN1-15). Witnessing patient suffering requires continuous psychological effort to manage the emotional reality while maintaining a professional role (RN12-15). This stress is compounded by frequent exposure to verbal or physical aggression from cognitively impaired individuals, which threatens the nurses’ psychological safety (RN4-17, RN19, RN21):

Patients’ aggressiveness causes difficulties when it is impossible to control their actions; they behave disrespectfully and cause fear and stress for both staff and nearby patients. (RN12)

They can be unpredictable and dangerous, so sometimes I fear for my own safety. (RN15)

Nurses experienced patient deaths as events connected to an accumulation of grief, emotional labor, and silent helplessness due to long-term relational bonds with geriatric patients (RN8-19). Nurses noted that the operational workflow leaves limited room for mourning, requiring them to suppress negative emotions and return immediately to care tasks, which they associated with long-term emotional exhaustion:

I react badly to the death of a patient. My thoughts run wild. Sometimes for two or three days I think and think. (RN8)

I feel psychological stress when a patient dies, especially when the death is sudden. Although I reassure myself that this is my job and I work with elderly people, I still don’t really manage to cope with negative emotions. (RN19)

Nurses noted that unaddressed clinical grief leads to emotional detachment as an involuntary coping mechanism against frequent mortality (RN1-10), while a lack of space for bereavement intersects with burnout and role alienation (RN11-21). Participants observed that high task volumes prevent meaningful communication with isolated patients, causing internal stress, ethical guilt, and a perception of professional failure due to conflicts with core nursing values (RN6-10, RN12, RN16-18, RN20). These lived experiences highlight how systemic pressures fracture therapeutic relationships, a strain compounded by interpersonal friction with patient relatives:

I have a great desire to communicate with patients, but we don’t have enough time, and it has a psychologically negative effect, as if I were avoiding them. (RN10)

The patient’s relatives do not hesitate to speak in a raised tone, offensively. And we have to hear what position they hold and how many influential acquaintances they have. Such things hurt a lot and remain in the memory and feelings for a long time. (RN12)

Nurses noted that continuous exposure to external hostility erodes professional dignity, making the workplace feel emotionally unsafe and contributing to anticipatory anxiety across shifts (RN1-9, RN10-21). Operational tension arises from family demands that disregard realistic physical and moral boundaries, such as demanding a continuous bedside presence or insisting on unverified medications (RN2-10). These expectations are experienced as expressions of distrust, which nurses associate with a defensive posture during interactions with relatives (RN3-16):

The demands of patient relatives do not always correspond to healthcare possibilities; often they are much higher than what is physically or morally possible. There have been moments when they ask to take care of their father around the clock without leaving the ward, or ask to include drugs of unknown origin in the treatment. (RN3)

Unrealistic demands from patients’ relatives indicate a lack of empathy and sensitivity toward nursing staff. For us, this is always an experience of distrust and humiliation. We must always be ready for a defensive position, making constant tension and stress daily companions of our work. (RN11)

Nurses noted that psychosocial exhaustion involves family behaviors that cross boundaries into explicit disruption, hostility, and mistrust (RN4-12, RN14, RN16, RN20). When families struggle to accept age-related decline, they project their distress onto staff through controlling demands and constant accusations that drive relational conflict and career fatigue (RN 4-16):

It is very difficult: hostile, dissatisfied with care, and there are various situations. Relatives' involvement becomes excessive, controlling, or disruptive, leading to conflict and mistrust of nurses as they cross the line from advocacy into demanding. (RN4)

The most difficult thing is that relatives do not always understand or adequately accept changes in their loved one’s health condition. Every time I experience a new feeling of tension and humiliation, which leads to the thought that I'm tired and I don't like this kind of work. (RN 12)

Nurses noted that the intersection of baseline alertness, unaddressed grief, behavioral outbursts, and family hostility creates an unsustainable psychosocial environment that compromises emotional well-being (RN1-21).

## Discussion

This study describes the dimensions of job dissatisfaction among elderly care nurses using five distinct categories identified through latent qualitative content analysis. Navigating work overload captures quantitative and temporal constraints, while confronting role overload reflects professional boundary dilution and scope expansion. Enduring an unfavorable practice environment mirrors macro-structural elements like managerial detachment and resource deficits, whereas surviving physical exhaustion indicates the impact of somatic and biomechanical tolls. Finally, bearing psychosocial exhaustion accounts for psychological and relational friction. These defined boundaries ensure that each category provides a structured, non-overlapping taxonomy of nurse job dissatisfaction.

### Navigating Work Overload

The experiences described by the nurses show how job dissatisfaction connects to work overload. Interpreted through the Job Demands-Resources model, high task volumes without sufficient organizational support deplete energy, leading to burnout (Bakker & Demerouti, 2017). This operational reality introduces administrative barriers and time scarcity that displace patient care, creating a conflict between professional goals and actual work conditions (Spector, 1988). These structural constraints reduce practice to a mechanical checklist that limits the nurse’s ability to engage in the foundational phases of Peplau’s (1952, 1991) therapeutic relationship, mirroring observations that compressed care environments limit clinicians from providing quality geriatric care (Aiken et al., 2012).

Similarly, scheduling irregularities and a lack of rest intersect with lower job satisfaction, echoing international findings (Dall’Ora et al., 2016). Viewed through the Demand-Control-Support model, high operational demands combined with limited control and low support increase workplace strain (Karasek, 1979). Under King’s (1981, 1992) framework, these time constraints impose an operational strain within the personal system that destabilizes the interpersonal system, where communication and transaction rely on mutual goal alignment. When workloads impede meaningful communication, the capacity to achieve consensus and attain desired outcomes becomes limited, aligning with evidence that work overload relates to lower satisfaction with the practice environment (Lesauskaite et al., 2006).

### Confronting Role Overload

The narratives regarding role overload highlight how job dissatisfaction connects to professional boundary changes and identity shifts within the care setting. Boundary confusion and identity dilution occur when nurses must absorb low-skill auxiliary duties or act as a corrective backup for colleagues due to performance gaps (Gribačiauskaitė & Žilinskienė, 2024). These descriptions underscore how unsupportive supervision, low compensation, and a lack of role recognition relate to career fatigue and heightened turnover intentions (Gao et al., 2025; Han et al., 2022). This structural friction alters the role clarity necessary for a functioning environment, disrupting Peplau’s (1991) emphasis on maintaining distinct professional identities to manage care effectively (Galanis et al., 2023; Ge et al., 2021).

King’s (1981) conceptualization of the social system illuminates this mismatch by explaining how authority, status, and role functions operate within an organization. In this study, the administrative layout fails to preserve the distinct status of the nursing role. When clinicians must perform non-nursing tasks to maintain safety, expectations conflict with actual practice, distorting interpersonal transactions. This necessity to compensate for operational deficits relates directly to physical strain, mirroring the low satisfaction described by nurses when forced to navigate structural limitations (Foong et al., 2021; Jakobsson Larsson et al., 2024).

### Enduring Unfavorable Practice Environment

An unfavorable practice environment shapes job dissatisfaction through unsupportive leadership, operational isolation, and low salaries that create a mismatch in organizational reciprocity (Galanis et al., 2023; Ge et al., 2021). Rigid supervision and inadequate pay relate to chronic burnout and voluntary turnover intentions, worsening regional workforce imbalances (Gao et al., 2025; Han et al., 2022; McHugh et al., 2011; OECD/European Commission, 2024). These organizational failures collide with ergonomic deprivation; executing manual transfers without mechanical lifters causes somatic strain, matching global data on equipment deficits (Harrad & Sulla, 2018; Jakobsson Larsson et al., 2024; Kumlin et al., 2020). This physical workload intersects with caring for individuals with behavioral symptoms of dementia, where time scarcity and cognitive care demands amplify moral distress and alter the structure of care (Foong et al., 2021; Kang & Bang, 2024; Kang & Hur, 2021; Kim & Lee, 2020).

Navigating these material limitations is an exercise in professional survival driven by a rigid administrative framework rather than the intrinsic challenges of clinical care (Gribačiauskaitė & Žilinskienė, 2024; Matulevičiūtė & Balžekienė, 2016). This structural environment restricts practice autonomy, reflecting historical and gendered expectations in nursing development (Karosas & Riklikiene, 2008). Viewed through King’s (1981) social system framework, the institutional layout fails to accommodate workforce needs. This unsupportive structure alters the interpersonal dynamics described by Peplau (1991), where an emphasis on compliance over empathy creates a sense of isolation. A lack of institutional validation forces nurses into a defensive posture during family interactions, ensuring that the emotional labor of managing patient aggression and processing clinical grief remains unmitigated, driving career fatigue.

### Surviving Physical Exhaustion

The experiences described by the nurses show how job dissatisfaction connects to physical exhaustion when managing frail older adults (Oliver, 2024). This bodily strain corresponds with cross-sectional data relating high task volumes and unfavorable shift patterns to employee discontent (Senek et al., 2020). When a practice environment presents limitations in support staff and lifting equipment, a conflict emerges between operational expectations and ergonomic safety, driving burnout, turnover intentions (Poku et al., 2022; Suh & Kim, 2019; Tuckett et al., 2014), and broader workforce migration (OECD, 2025).

These constraints reflect top-down administrative designs, such as the historical Semashko model, which require a transformation of organizational culture across post-Soviet contexts (Sheiman et al., 2018; Tiittanen et al., 2021). Treat-ing a specialized clinical role as unsupported manual labor accelerates physical exhaustion and underscores how policy configurations underemphasize physical health and professional dignity.

These themes relate directly to the personal system within King’s (1981) framework, where physical discomfort alters the nurse’s perception of workplace safety and drives work discontent. This somatic depletion intersects with Peplau’s (1952) perspective that the nurse must function as an effective relational instrument. Continuous physical exertion without physiological reset limits energy reserves, restricting the capacity to engage empathetically and sustain the intensive interpersonal commitment required for care.

### Bearing Psychosocial Exhaustion

The experiences described by the nurses show how job dissatisfaction connects to psychosocial exhaustion and constant emotional labor. Operating under a baseline of mental alertness regarding safety introduces psychological pressure, mirroring data that link high task demands to nurse dissatisfaction (Senek et al., 2020). Witnessing severe suffering and degenerative decline triggers helplessness and moral distress when standard protocols cannot provide comfort (Suh & Kim, 2019). This high emotional output, combined with a lack of managerial recognition, relates directly to burnout, voluntary turnover intentions, and missed care (Poku et al., 2022; Tuckett et al., 2014; White et al., 2019).

This internal strain is compounded by exposure to patient aggression and unprocessed grief. Managing unpredictable outbursts from cognitively impaired individuals depletes emotional reserves and induces severe environmental stress (Wang & Nguyen, 2023). Furthermore, because operational workflows provide limited space for mourning after a patient dies, nurses must suppress negative emotions and return immediately to care tasks, driving role alienation and burnout (Wudarczyk et al., 2025; Zhang et al., 2022).

Viewed through King’s (1981) framework, these hostile family expectations and verbal disruptions compromise the interpersonal system by inducing anticipatory anxiety. This strain intersects with Peplau’s (1991) relational perspective on processing clinical grief. When workflows force the immediate suppression of negative emotions, the lack of bereavement space triggers emotional detachment as an involuntary coping mechanism, transforming intensive emotional labor into role alienation and career fatigue.

### Model Development

**Figure 1.**
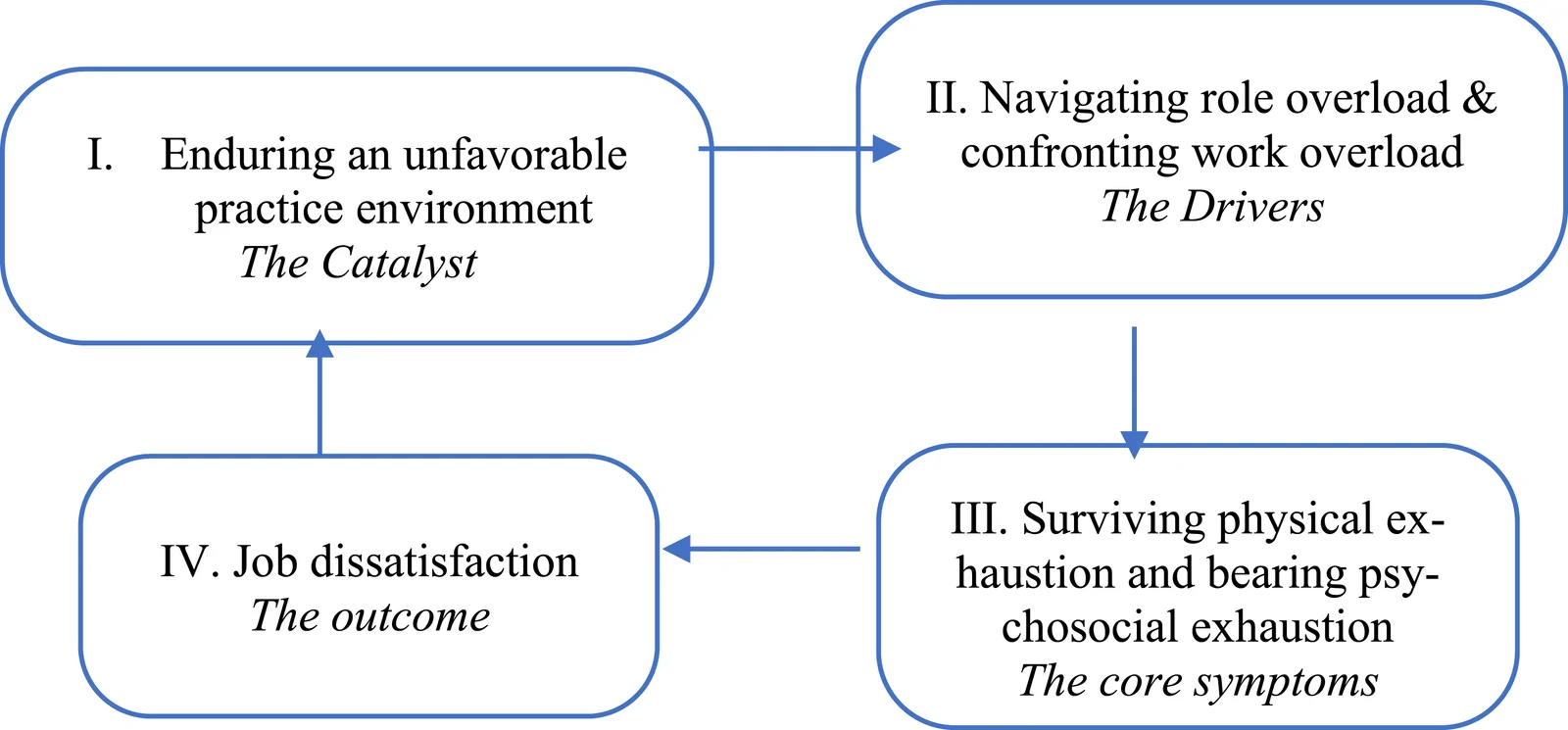
The cyclic loop model of nurse dissatisfaction in elderly care while working in hospital inpatient settings. *Note.* Developed and compiled entirely by the authors based on empirical study findings

The cyclic loop model of nurse dissatisfaction in hospital inpatient elderly care (see Figure 1) was developed from the empirical categories and subcategories presented in Table 3. Utilizing latent qualitative content analysis (Graneheim & Lundman, 2004), the five initial manifest dimensions were synthesized into four processual stages. This structural reduction was necessitated by data indicating that quantitative task demands (“Navigating work overload”) and qualitative boundary tensions (“Confronting role overload”) are experienced concurrently during a shift, operating as intertwined forces in Stage II: The Drivers rather than separate linear steps.

The proposed model establishes an ontological position that modifies the linear trajectories of classical nursing theories. It contrasts with Peplau’s (1997) sequential phases of interpersonal anxiety reduction and King’s (1981) reciprocal transactions for mutual goal-setting. The empirical accounts suggest that under-resourced acute geriatric environments present systemic operational barriers that restrict the clinician’s capacity to maintain therapeutic interactions, thereby anchoring nurses within an organizational loop. This process expands Racy et al. (2021) and Naegle et al. (2023) macro-level staffing paradigms by linking objective resource deficits with Hochschild’s (2012) construct of emotional labor. Manifestations such as “compulsory absorption of auxiliary tasks” and “sustained hyper-vigilance” intersect with geriatric-specific pressures, including “compassion fatigue from exposure to suffering” and “cumulative grief from resident mortality.” Within this qualitative context, job dissatisfaction operates not as a static terminal outcome, but as an active interpretive feedback lens that reinforces the negative perception of the baseline practice environment:

#### Stage I: Enduring an unfavorable practice environment

The model positions the unfavorable practice environment as the initial operational catalyst where institutional resource scarcity intersects with complex geriatric care demands. Participants highlighted an authoritarian management structure and a fractured effort-reward balance, leaving clinicians feeling undervalued (Ge et al., 2021; Zhang et al., 2022), unsupported (Li et al., 2024; Poku et al., 2022), and ill-equipped (Gao et al., 2025). This institutional deficit limits the foundational macro-organizational baseline necessary for safe care trajectories (Racy et al., 2021; Naegle et al., 2023), colliding with the multidimensional demands of older populations (Abdi et al., 2019). These environmental restrictions force a state of professional survival that aligns with literature linking poor staffing to high turnover intentions (Aiken et al., 2012; Aiken et al., 2021; Dall’Ora et al., 2016) and exhausted coping reserves (Bakker & Demerouti, 2017). Within the Lithuanian framework, this systemic reality restricts the initial social conditions described in King’s (1981) system, diminishing nurse self-efficacy under top-down administrative constraints (Blaževičienė et al., 2016; Dasgupta, 2013; Deksnytė et al., 2023).

#### Stage II: Navigating work overload and confronting role overload

Stage II illustrates how navigating work overload and confronting role overload converge within a restrictive environment of unmanageable patient volumes and fixed time constraints. The transcripts expose a fragmented workflow driven by chronic work-rest imbalances, intensive documentation, indistinct job boundaries, and the necessity of performing non-nursing tasks. In nursing science, these barriers operate as hindrance stressors that accelerate pacing and drive care omissions (White et al., 2019), diluting specialized roles into low-skill labor (Gao et al., 2025; Jun, Park, & Rosemberg, 2022). This structural trap suppresses the open interpersonal transactions defined by King (1981), rendering mutual goal-setting operationally difficult. Furthermore, this friction forces the compulsory absorption of auxiliary tasks and sustained hyper-vigilance, operationalizing Hochschild’s (2012) construct of emotional labor. These constraints cause severe moral distress and an erosion of professional meaning, as acute time poverty prevents safe care, generating guilt (Kang & Bang, 2024; Kang & Hur, 2021) and violating core professional values (Heydari et al., 2019; Li & Chen, 2023). This ethical strain is pronounced when managing complex clinical trajectories or cognitive decline (Kim & Lee, 2020; Kumlin et al., 2020), transforming the environment into a source of professional alienation that expands Racy et al. (2021) and Naegle et al. (2023) staffing paradigms beyond simple mathematical ratios (McHugh et al., 2011; Poku et al., 2022).

#### Stage III: Surviving physical exhaustion and bearing psychosocial exhaustion

Surviving physical exhaustion and bearing psychosocial exhaustion function as core clinical symptoms arising from high patient dependency, repetitive manual lifting, and prolonged working hours. Geriatric nurses operate under an unrelenting kinetic and emotional load characterized by chronic musculoskeletal strain, static postures, and an unyielding volume of hands-on care demands. This depletion represents the advanced stage of Hochschild’s (2012) emotional labor paradigm, where managing feelings under high-stakes conditions—such as exposure to aggressiveness, frequent resident deaths, and family pressure—leads to systemic self-alienation (Kumlin et al., 2020; Marinho et al., 2022; Wang & Nguyen, 2023).

This exhaustion disrupts the professional baseline assumed in Peplau’s (1997) interpersonal relations model, as the clinician’s internal coping mechanisms fail under acute resource scarcity, preventing them from maintaining a therapeutic presence (White et al., 2019). When structurally isolated during understaffed night shifts, the tension of maintaining hyper-vigilance transforms observation into a source of somatic and psychological distress (Suh & Kim, 2019), leaving the workforce in a permanent depletion that compromises their quality of life (Blaževičienė et al., 2016). This exhaustion compromises clinical care trajectories, as described by Racy et al. (2021) and Naegle et al. (2023) quality metrics, by depriving vulnerable older adults of safe, stable, and structured care (Foong et al., 2021). Clinician exhaustion is an outcome of a rigid structure that treats human health as an expandable commodity (McHugh et al., 2011; Poku et al., 2022).

#### Stage IV: Job dissatisfaction

The final stage establishes job dissatisfaction as the outcome when the operational workplace reality fails to meet professional expectations, representing an interruption of Peplau’s (1997) expected professional resolution phase and King’s (1981) purposeful social systems. This outcome functions not as a static terminal endpoint, but as an active feedback mechanism that reinforces Stage I by lowering the clinician’s threshold of tolerance for the unfavorable practice environment, continuing the self-reinforcing loop.

This decline in professional well-being drives high nurse turnover, threatening workforce stability as clinicians view unit or professional abandonment as a mechanism for survival (Dasgupta, 2013; Jakobsson Larsson et al., 2024). This operational attrition restricts clinical safety margins, accelerates care omissions, and alters the baseline capacity for holistic patient advocacy. It reflects the advanced phase of Hochschild’s (2012) emotional labor paradigm, where prolonged management of feeling under unsupportive conditions results in professional estrangement and a break in identity (Kim & Lee, 2020).

Furthermore, this attrition represents a structure where job dissatisfaction functions as a predictable response to an underfunded administrative layout that treats workforce safety as an expandable commodity. This structural breakdown aligns with Racy et al. (2021) and Naegle et al. (2023) analyses of macro-organizational metrics on clinical quality, while marking the disruption of the reciprocal transactions required in King’s (1981) framework. Consequently, instead of achieving the progressive professional resolution phase outlined by Peplau (1997), clinicians face compounding systemic pressures that exacerbate work dissatisfaction and somatic symptoms during subsequent cycles (Galanis et al., 2023; Senek et al., 2020; Tuckett et al., 2014).

### Strengths and Limitations

The qualitative approach allowed nurses to express the emotional burden of elderly care and highlighted region-specific factors. The eligibility criteria established a homogenous, specialized cohort capable of providing deep insights into job dissatisfaction dynamics (Elo et al., 2014). Selecting university-educated nurses with over a decade of specialized experience isolated systemic and operational drivers, minimizing confounding variables like entry-level transition shock or differing educational tracks. This focus supports transferability by offering a context-specific model for comparable healthcare settings.

However, several limitations must be noted. Excluding nurses with diplomas from non-university higher education institutions (kolegijos) leaves the experiences of those professionals unexamined. Similarly, requiring a minimum of 10 years of clinical experience excluded early-career nurses, meaning initial entry-level attrition and transition shock remain unexamined.

Socioculturally, the historical undervaluing of the nursing role as subordinate to medicine, coupled with a weak research culture and low professional prestige, can hinder open discussion. Finally, the exclusively female composition of the cohort presents limitations regarding gender bias and transferability. Nursing in Lithuania remains a female-dominated profession, with men constituting less than 1% of the workforce, intertwining the discipline with traditional societal expectations of caregiving and historical post-Soviet occupational structures (Gribačiauskaitė & Žilinskienė, 2024; Karosas & Riklikiene, 2008). This sample reflects national demographic realities rather than restrictive study criteria, meaning the findings may not fully represent the experiences of male nurses or be broadly transferable to more gender-diverse healthcare systems (Gribačiauskaitė & Žilinskienė, 2024).

### Implications for Practice

The interpretive cyclic model of nurse dissatisfaction in hospital inpatient elderly care provides a processual perspective for clinical practice, suggesting that nurse dissatisfaction is perceived not as a static terminal outcome but as a recurring, self-reinforcing process. In the context of inpatient geriatric care—where clinical recovery trajectories are often prolonged and routine tasks may be experienced as monotonous—this interpretive model offers an alternative framework for nursing administration. The narratives indicate that addressing job dissatisfaction and its associated somatic and psychosocial outcomes involves proactive, tailored strategies aligned with the specific phases described by participants, rather than generic organizational interventions.

For nursing managers, the shared experiences highlight the importance of recognizing how clinicians navigate these interconnected phases, allowing for the implementation of supportive structures that address both structural workloads and emotional labor boundaries. Furthermore, the findings encourage elderly care nurses to engage in continuous practice reflection, examining routine, taken-for-granted care processes. Such professional reflection is positioned as a pathway that may assist nurses in transitioning from a state of passive dissatisfaction toward a constructive, adaptive phase, facilitating the re-learning of clinical approaches and the reconstruction of professional meaning within the workplace.

## Conclusion

Understanding nurse job dissatisfaction involves looking beyond standardized surveys. Narrative descriptions shared by experienced nurses capture operational and socio-cultural parameters in inpatient elderly care wards. The participants’ experiences are synthesized into an interpretive model illustrating that job dissatisfaction is experienced as a process rather than a static outcome. The model illuminates how an unfavorable environment intersects with workload and role overloads alongside exhaustion for elderly care nurses. This experience functions as a lens that worsens baseline perceptions, showing that the retention of elderly care nurses involves comprehensive rather than isolated interventions. This interpretation provides experience-based insights for nursing leadership to target the cyclic feedback mechanism through supportive structures and active modifications to the daily elderly care nursing environment.

## Supplemental Material

Supplemental Material - Job Dissatisfaction Among Elderly Care Nurses Working in Hospital Inpatient Settings: A Qualitative Study Supplemental Material for Job Dissatisfaction Among Elderly Care Nurses Working in Hospital Inpatient Settings: A Qualitative Study by Vilma Žydžiūnaitė, Marija Truš, Indrė Brasaitė-Abromė in Sage Open Nursing.

Supplemental Material - Job Dissatisfaction Among Elderly Care Nurses Working in Hospital Inpatient Settings: A Qualitative Study Supplemental Material for Job Dissatisfaction Among Elderly Care Nurses Working in Hospital Inpatient Settings: A Qualitative Study by Vilma Žydžiūnaitė, Marija Truš, Indrė Brasaitė-Abromė in Sage Open Nursing.
