# Supplementary material for: Job Dissatisfaction Among Elderly Care Nurses Working in Hospital Inpatient Settings: A Qualitative Study
Source: SAGE Open Nurs. 2026 Aug 24;12:23779608261478192. doi: 10.1177/23779608261478192 (PMC13504132; doi:10.1177/23779608261478192)
Supplement: Supplementary file 1 [file sj-pdf-1-son-10.1177_23779608261478192.pdf]

Supplementary File 1: COREQ checklist

Consolidated criteria for reporting qualitative studies (COREQ): 32-item checklist

Developed from:

Tong, A., Sainsbury, P., & Craig, J. (2007). Consolidated criteria for reporting qualitative research (COREQ): a 32-item checklist for interviews and focus groups. International Journal for Quality in Health Care, 19(6), 349 – 357. http://dx.doi.org/10.1093/intqhc/mzm042

Manuscript Title: Job Dissatisfaction among Elderly Care Nurses Working in Hospital Inpatient Settings: A Qualitative Study

Corresponding Methodology: Latent Qualitative Content Analysis (Graneheim & Lundman, 2004)

**Item No**	**Guide Questions/Description**	**Description**
**Domain 1: Research team and reflexivity**		
**Personal Characteristics**		
1. Interviewer/ facilitator	Which author/s conducted the interview or focus group?	Interviews were conducted collaboratively by all three authors.
2. Credentials	What were the researcher's credentials? E.g., PhD, MD	All three researchers hold PhD degrees (Doctor of Health Sciences, Nursing).

Item No	Guide Questions/Description	Description
3. Occupation	What was their occupation at the time of the study?	All three are female academic nursing scholars and university professors whose professional activities are confined exclusively to higher education and academic research.
4. Gender	Was the researcher male or female?	Female (all three researchers).
5. Experience and training	What experience or training did the researcher have?	All three researchers have extensive post-doctoral training in qualitative research methodologies, semi-structured interviewing, and latent qualitative content analysis.
**Relationship with participants**		
6. Relationship established	Was a relationship established prior to study commencement?	No relationship was established prior to the study. Participants were approached via institutional access networks and formal invitations
7. Participant knowledge of the interviewer	What did the participants know about the researcher? e.g. personal goals, reasons for doing the research?	Participants were informed that the interviewers were purely academic researchers from the university, independent of clinical management, who aimed to explore workplace strain to build a nursing workforce model.
8. Interviewer characteristics	What characteristics were reported about the interviewer/facilitator? e.g. Bias, assumptions, reasons and interests in the research topic	The researchers were critical, empathetic scholars with former clinical nursing backgrounds. Potentially biased assumptions regarding workplace strain were managed via systematic reflexivity, independent parallel coding, and bracketing protocols.
**Domain 2: study design**		
**Theoretical framework**		

Item No	Guide Questions/Description	Description
9. Methodological orientation and Theory	What methodological orientation was stated to underpin the study? e.g. grounded theory, discourse analysis, ethnography, phenomenology, content analysis	Qualitative descriptive approach utilizing the thematic and architectural framework of latent qualitative content analysis (Graneheim & Lundman, 2004)
**Participant selection**		
10. Sampling	How were participants selected? e.g., purposive, convenience, consecutive, snowball	Purposive sampling.
11. Method of approach	How were participants approached? e.g., face-to-face, telephone, mail, email	Formal written recruitment letters and participant information sheets distributed through university networks to inpatient wards.
12. Sample size	How many participants were in the study?	Total sample consisted of 21 participants (N = 21).
13. Non-participation Setting	How many people refused to participate or dropped out? Reasons?	No selected participants dropped out or officially refused to participate once contact was successfully made.
14. Setting of data collection	Where was the data collected? e.g., home, clinic, workplace	Hospital inpatient elderly care units across Lithuania's five largest cities (Vilnius, Kaunas, Klaipėda, Šiauliai, Panevėžys).
15. Presence of nonparticipants	Was anyone else present besides the participants and researchers?	No non-participants were present during the interviews; only the individual participant and one assigned researcher were present.
16. Description of sample	What are the important characteristics of the sample? e.g. demographic data, date	All 21 participants were licensed female nurses with a mean age of 45.52 years, holding a university Bachelor's degree (nine also held Master's degrees), a mean total hospital experience of 20.43 years, and an average of 17.39 years specific to elderly care.
**Data collection**		

Item No	Guide Questions/Description	Description
17. Interview guide	Were questions, prompts, and guides provided by the authors? Was it pilot tested?	A validated, semi-structured interview guide with five introductory (manifest) and five follow-up (latent) prompts was used (see Additional File 1). The tool underwent content validation by an expert panel (three professors) and face validation/pilot testing with two eligible nurses (excluded from final N=21 sample).
18. Repeat interviews	Were repeat interviews carried out? If yes, how many?	No repeat interviews were conducted; each participant completed a single narrative session lasting 56–194 minutes.
19. Audio/visual recording	Did the research use audio or visual recording to collect the data?	All interviews were recorded using local digital audio recorders with full participant consent. Files were immediately transferred to secure university servers and deleted from the devices.
20. Field notes	Were field notes made during and/or after the interview or focus group?	Extensive field notes capturing contextual observations, emotional nuances, and immediate reflexive thoughts were compiled by each interviewer immediately post-interview.
21. Duration	What was the duration of the interviews or focus group?	The duration of the interviews ranged from approximately 56 to 194 minutes.
22. Data saturation	Was data saturation discussed?	Inductive category saturation was operationalized using the framework of Saunders et al. (2018). Preliminary category saturation was achieved by the 17th interview. Four additional validation interviews (18–21) were conducted to confirm conceptual and structural redundancy; no new subcategories or categories emerged, and recruitment was concluded.
23. Transcripts returned	Were transcripts returned to participants for comment and/or correction?	Transcripts were not returned to participants, as the focus was on extracting inductive cross-case latent meaning rather than individual member-checking of raw manifest data.

Item No	Guide Questions/Description	Description
**Domain 3: analysis and findings**		
**Data analysis**		
24. Number of data coders	How many data coders coded the data?	All three researchers were actively involved in data analysis. Open coding of the first five transcripts was performed independently before joint analysis.
25. Description of the coding tree	Did the authors provide a description of the coding tree?	The coding structure followed Graneheim and Lundman's (2004) framework. Open codes were abstracted into subcategories, grouped into six interconnected categories, and synthesized into one overarching main latent theme (the cyclic, self-reinforcing loop model of nurse dissatisfaction).
26. Derivation of themes	Were themes identified in advance or derived from the data?	Derived inductively from the raw data text using classic, tactile manual paper-and-pen configuration grids. No automated text software or AI tools were used.
27. Software	What software, if applicable, was used to manage the data?	No computer software or AI was used. Analysis was strictly human-driven and performed manually to protect text context, emotional nuances, and authentic regional idioms.
28. Participant checking	Did participants provide feedback on the findings?	Participants did not check transcripts or categories. Methodological trustworthiness was instead maintained via regular, 100% mutual consensus-building sessions among the three researchers to resolve analytical disagreements.
**Reporting**		
29. Quotations presented	Were participant quotations presented to illustrate the themes/findings? Was each quotation identified? e.g., participant number	Direct, fully anonymized participant quotations are presented throughout the Results section to substantiate each developed category and subcategory.

Item No	Guide Questions/Description	Description
30. Data and findings consistent	Was there consistency between the data presented and the findings?	There is clear, documented consistency between the raw meaning units, condensed text, and the abstracted subcategories reported.
31. Clarity of major themes	Were major themes clearly presented in the findings?	There is clear, documented consistency between the raw meaning units, condensed text, and the abstracted subcategories reported.
32. Clarity of minor themes	Is there a description of diverse cases or a discussion of minor themes?	Minor variations, contextual structural strains (such as labor migration and distinct cultural expectations), and specific subcategory adjustments are clearly integrated and discussed within the findings.
