# Supplementary material for: Job Dissatisfaction Among Elderly Care Nurses Working in Hospital Inpatient Settings: A Qualitative Study
Source: SAGE Open Nurs. 2026 Aug 24;12:23779608261478192. doi: 10.1177/23779608261478192 (PMC13504132; doi:10.1177/23779608261478192)
Supplement: Supplementary file 2 [file sj-pdf-2-son-10.1177_23779608261478192.pdf]

Semi-Structured Interview Guide

Project Title: Job Dissatisfaction among Elderly Care Nurses Working in Hospital Inpatient Settings: A Qualitative Study

Methodological Design: Qualitative descriptive approach utilizing latent qualitative content analysis (Graneheim & Lundman, 2004).

Purpose of the Tool: This interview guide employs an open-ended, two-tiered architecture. The Introductory Questions anchor the conversation in the socio-operational reality of the inpatient ward (manifest content), while the Follow-up Questions dynamically prompt the participant to look past surface-level descriptions to explore underlying psychological, behavioral, and professional-identity meanings (latent content).

Part I: Pre-Interview Demographic & Eligibility Verification

Before commencing the narrative audio recording, the researchers manually documented the following structural parameters to confirm adherence to the inclusion criteria:

Participant Alphanumeric Code (e.g., Participant 01): ______________

Current City of Employment (Lithuania): ______________

Total Years of Hospital Inpatient Experience (Minimum 10): ______________

Total Years of Specific Experience in Elderly Care (Minimum 10): ______________

Highest Nursing Educational Attainment (University Bachelor's/Master's): ______________

Part II: Part II: Core Semi-Structured Interview Protocol and Methodological Substantation

Introductory Questions (Socio-Operational Reality / Manifest)	Follow-up Questions (Latent Meaning Extraction / Probes)	Methodological Substantiation (Alignment with Study Issue & Aim)
1. Can you describe a recent situation at work that made you feel frustrated, tense, or unsupported?	* What was going through your mind at that moment?	Issue Alignment: Targets the unfavorable practice environment component of the cycle. It allows highly experienced nurses to ground their answers in recent, concrete clinical realities.<br>Aim Alignment: Generates the manifest narrative foundation necessary to map out how daily organizational breakdowns act as the initial, active triggers driving the broader cycle of dissatisfaction.
2. When you think about your daily work, what aspects of your role cause you stress?	* What did you do in response to that feeling?	Issue Alignment: Directly explores the physical and operational burdens specific to inpatient geriatric care (e.g., heavy manual lifting of immobile patients, high patient-to-nurse ratios, cognitive decline).<br>Aim Alignment: Unpacks the behavioral coping adjustments and active responses of the clinicians, tracking how work and role overload directly feed into physical and psychosocial exhaustion.
3. Describe a time when you felt you couldn't provide the quality of care you wanted to. What was happening?	* Why do you think that situation made you feel that way?	Issue Alignment: Explores the fundamental conflict between professional/ethical nursing ideals and the structural limits of modernized yet short-staffed post-Soviet healthcare systems facing high migration rates.<br>Aim Alignment: Extracts the attributional and root-cause reflections of the nurses, pinpointing the exact operational imbalances (such as excessive bureaucratic paperwork) that trigger emotional exhaustion.
4. What makes you think about difficulties in your current position in elderly care while working in an inpatient unit at the hospital?	* How did that experience make you feel about your role as a nurse?	Issue Alignment: Investigates the specialized stressors unique to the inpatient geriatric environment, separating this context from primary care or general medical-surgical settings.<br>Aim Alignment: Evaluates professional identity erosion and motivation decline, demonstrating the latent stage where the gap between high expectations and daily operational reality transforms into chronic job dissatisfaction.
5. How does the work culture and management on your unit influence your day-to-day satisfaction?	* Can you help me understand that a little better?	Issue Alignment: Addresses the socio-cultural matrix, regional institutional pressures, and the presence or lack of managerial and teamwork support systems.<br>Aim Alignment: Uncovers the temporal, feedback-driven mechanism of the loop, detailing how aggregated daily dissatisfaction and motivation erosion ultimately feed back into and further degrade the unit's overall practice environment.

Part III: Closing and Reflexive Field Protocol

- Concluding Narrative Check: Before concluding, the participant is systematically asked: "Is there anything else regarding your daily tasks, regional challenges, or your lived experiences of job dissatisfaction that we haven't covered today?"
- Post-Interview Action: Immediately following the interview, the recording device is turned off. In accordance with the study's trustworthiness and bracketing protocols, the individual researcher compiles extensive field notes tracking immediate contextual details, vocal

changes, and emotional nuances to manage personal assumptions prior to collaborative data transcription
